# Factors Associated With the Severity of Diabetic Ketoacidosis on Admission in Pediatric Intensive Care: A Retrospective Study

**DOI:** 10.7759/cureus.76460

**Published:** 2024-12-27

**Authors:** Mahamoud Ahmed Houssein, Manira Moussa Ahmed, Zineb Serhier, Samali El Mehdi, Karim El Aidaoui, Jihane Ziati, Chekhlabi Nabila, Nezha Dini, Chafik El Kettani, Amal Haoudar

**Affiliations:** 1 Anesthesia and Critical Care, Cheikh Khalifa International University Hospital, Mohammed VI University of Health Sciences, Casablanca, MAR; 2 Pediatrics, Cheikh Khalifa International University Hospital, Mohammed VI University of Health Sciences, Casablanca, MAR; 3 Laboratory of Medical Informatics, Hassan II University, Casablanca, MAR; 4 Laboratory of Clinical Neuroscience and Mental Health, Hassan II University, Casablanca, MAR; 5 Anesthesia and Critical Care, Mohammed V Military Training Hospital, Mohammed V University, Rabat, MAR

**Keywords:** children, diabetes type 1, pediatric intensive care, severity, ketoacidosis

## Abstract

Background

Diabetic ketoacidosis (DKA) is one of the leading causes of morbidity and mortality in children with diabetes, often requiring intensive care unit management. This study aimed to identify factors associated with the severity of DKA in infants and children hospitalized in pediatric intensive care.

Methodology

This retrospective, monocentric, descriptive, analytical study included infants and children aged one month to 17 years who presented with DKA meeting the International Society for Pediatric and Adolescent Diabetes 2022 criteria. The study was conducted at the Pediatric Intensive Care Unit of the International University Hospital Cheikh Khalifa in Casablanca from July 2018 to February 2023. For data analysis, patients were divided into two groups, namely, severe DKA and non-severe DKA. Data analysis was performed using Jamovi software.

Results

Of the 63 children included, 26 (41.3%) had severe DKA, and 37 (58.7%) had non-severe DKA. The mean age was 8.92 ± 5.18 years, with a sex ratio of 0.97. Statistical analysis revealed a significant clinical difference between DKA severity and the presence of dyspnea at admission (p = 0.004) and drowsiness (p = 0.003). Regarding biological parameters, the study showed that patients with severe DKA had significantly higher white blood cell (WBC) counts (p = 0.013), as well as significantly higher procalcitonin (PCT) levels (p = 0.038) and C-reactive protein (CRP) concentrations (p = 0.011) compared to children admitted with non-severe DKA. No significant differences were observed between the two groups regarding age, sex, symptoms, triggering factors, or clinical outcomes.

Conclusions

The severity of DKA in children is associated with the presence of neurological disturbances and dyspnea at admission, as well as a significant elevation in WBC count, CRP, and PCT.

## Introduction

Diabetes is a significant public health issue worldwide, affecting more than 2 million people in Morocco, including approximately 15,000 children [1]. In children, acute complications of diabetes include diabetic ketoacidosis (DKA), hyperosmolar hyperglycemic state, hypoglycemia, and lactic acidosis. DKA is a potentially life-threatening emergency and is among the primary causes of morbidity and mortality in children with type 1 diabetes, although it can also occur in cases of type 2 diabetes [2,3]. Furthermore, DKA is the initial presentation of type 1 diabetes in 12.8% to 80% of cases [4,5]. The most severe complication associated with DKA is cerebral edema, which remains the leading cause of death in these patients [6,7].

The severity of DKA is classified according to the degree of acidosis as mild, moderate, or severe [8]. Given the preventable nature of this complication in pediatric populations, there is a crucial need for a deeper understanding of the factors influencing its severity. In Morocco, however, limited research has specifically addressed these determinants within pediatric cohorts. This study aims to identify the factors associated with the severity of DKA at admission among children.

## Materials and methods

Study overview

This retrospective, monocentric, descriptive, analytical study was conducted in the pediatric intensive care unit (PICU) of the Cheikh Khalifa International University Hospital in Casablanca over a five-year period from July 2018 to February 2023.

Target population

The inclusion criteria consisted of all patients hospitalized in the PICU for DKA, aged between 28 days and 17 years, who met the biological criteria established by the 2022 guidelines of the International Society for Pediatric and Adolescent Diabetes (ISPAD) [8]. These criteria were as follows: blood glucose levels greater than 2 g/L, the presence of ketonuria, and a pH below 7.3 and/or a bicarbonate (HCO_3_-) level below 18 mmol/L. Exclusion criteria included cases of hyperglycemia not meeting the ISPAD 2022 guidelines, as well as incomplete or untraceable medical records.

In accordance with the hospital protocol, all children and infants with ketoacidosis, whether severe or non-severe, are admitted to the PICU for monitoring. The anonymity of patients was maintained throughout the study.

Data collection

Clinical data, blood gas analysis, treatment, and patient outcomes were collected from patient medical records stored in the DxCare software system at the Cheikh Khalifa University Hospital. Biological data were retrieved through the Laboratory Information Management System.

The classification of DKA severity was based on the 2022 ISPAD guidelines. Mild DKA was defined by a venous pH <7.3 or serum bicarbonate <18 mmol/L, moderate DKA by a pH <7.2 or serum bicarbonate <10 mmol/L, and severe DKA by a pH <7.1 or serum bicarbonate <5 mmol/L [8]. To meet the study’s objective, patients were categorized into two groups, namely, severe DKA and non-severe DKA (the latter encompassing both moderate and mild cases), according to the 2022 ISPAD classification [8]. Severe DKA was defined as a pH <7.1 and/or bicarbonate <5 mmol/L, while non-severe DKA was characterized by a pH between 7.1 and 7.3 and/or bicarbonate levels between 5 and 18 mmol/L.

Data were recorded in a pre-prepared table and entered using Microsoft Excel 2016 (Microsoft Corp., Armonk, NY, USA). For patients who were hospitalized multiple times for DKA during the study period, only the first hospitalization was included for data collection.

The standardized therapeutic protocol in the unit consisted of vascular filling in cases of vascular collapse, rehydration, insulin infusion, correction of electrolyte disturbances, and treatment of underlying causes (triggering factors).

Statistical analysis

The data analysis was performed using Jamovi software. Quantitative data were described by the mean, standard deviation, median, and minimum and maximum values. Categorical data were expressed as frequencies and percentages. Comparisons of quantitative variables were performed using the non-parametric Mann-Whitney test and the Student’s t-test, while qualitative data were compared using the chi-squared (χ²) test. A p-value of less than 0.05 was considered statistically significant.

## Results

A total of 95 patients were hospitalized for DKA during the study period, of whom 63 met the inclusion criteria. The average age of the patients was 8.92 ± 5.18 years, ranging from one to 17 years, and the male-to-female ratio was 0.97. The age at diagnosis of diabetes was 5.7 ± 4.3 years, and the average weight was 28.35 ± 14.81 kg. The healthcare coverage status was recorded in 60 patients, of whom 91.7% (55 patients) had health insurance. The first episode of DKA was observed in 61.9% (39 patients) of the patients. A third of the patients (33.3%) had a family history of diabetes, and consanguinity was present in seven (11.1%) patients. Overall, 25 (39.7%) patients had previously experienced at least one episode of DKA.

The most common clinical symptoms included polyuria-polydipsia syndrome, observed in 41 (65.1%) patients, followed by dyspnea in 32 (50.8%) patients, weight loss and vomiting, each observed in 29 (46%) patients, and abdominal pain in 27 (42.9%) patients. Altered consciousness (drowsiness) was present in 19 (30.2%) patients at admission. Dehydration was found in 46 (73%) patients, with 60% of cases classified as moderate dehydration and 13.3% as severe dehydration. Tachycardia was observed in 40 (63.5%) patients, and cardiovascular collapse was noted in eight (12.7%) patients at admission. The average capillary blood glucose at admission was 4.72 ± 1.13 g/L, with 29 (46%) patients having a blood glucose level greater than 5 g/L. A urine test strip showed ketonuria with four crosses in seven (11.1%) patients, three crosses in 46 (73%) patients, and two crosses in 10 (15.9%) patients. The most common precipitating factor was infections, found in 22 (34.9%) patients, followed by dietary deviations in 15 (23.8%) patients, poor treatment adherence in 12 (19%) patients, and insulin discontinuation in nine (14.3%) patients.

In terms of arterial blood gas analysis, the average pH was 7.12 ± 0.15, bicarbonate (HCO_3_-) was 8.06 ± 4.8 mmol/L, PCO_2_ was 20.14 ± 11 mmHg, and PaO_2_ was 109.21 ± 29.8 mmHg. DKA was classified as severe in 41.3% (26 patients) and non-severe in 58.3% (37 patients). The average sodium (Na^+^) and potassium (K^+^) levels were 134.05 ± 5.67 mmol/L and 4.58 ± 1.1 mmol/L, respectively. Regarding renal function, the mean serum creatinine was 9.44 ± 4.73 mg/L, and the mean urea level was 0.35 ± 0.15 g/L. For the infectious workup, the average white blood cell (WBC) count was 19 ± 7.5 × 10³ cells/mm³, the C-reactive protein (CRP) level was 10.76 mg/L, and procalcitonin (PCT) performed in 45 (71.4%) patients, was positive in 28.9% of cases (13 patients). The mean glycated hemoglobin (HbA1c) level was 11.61% ± 1.5%, with values ranging from 8.7% to 16.3%. The evolution was favorable for all patients. The mean duration of insulin therapy in the PICU was 37.57 ± 18.96 hours. The average stay in the PICU was 3.44 ± 1.71 days, while the total hospital stay averaged 7.54 ± 3.45 days.

Analytical study

The average age was 11.79 ± 4.67 years for patients with severe DKA and 6.63± 4.41 years for those with non-severe DKA. The difference was not statistically significant (p = 0.915). Similarly, no significant difference was observed regarding sex (p = 0.700), medical insurance coverage (p = 0.109), or average weight (p = 0.877). Table 1 presents a comparison between severe and non-severe DKA.

**Table 1:** Comparison of severe and non-severe diabetic ketoacidosis. P-values were calculated by Student’s t-test or Mann–Whitney U test for continuous variables and the chi-squared (χ²) test for categorical variables. *: significant p-value; ^1^: expressed in mean ± standard deviation; ^2^: expressed in frequency (%). WBC: white blood cell count; PCT: procalcitonin; CRP: C-reactive protein; DKA: diabetic ketoacidosis; BUN: blood urea nitrogen; HbA1c: hemoglobin A1c; Na^+^: serum sodium; K^+^: serum potassium

Characteristics	Total (63)	Severe DKA (26)	Non-severe DKA (37)	P-value
Age (years)^1^	8.92 ± 5.18	11.79 ± 4.67	6.63 ± 4.41	0.915
Gender M/F^2^	31/32	13/13	18/19	0.700
Health insurance^2^	55 (91.7%)	24 (43.6%)	31 (56.4%)	0.109
Mean weight at presentation (kg)^1^	28.35 ± 14.81	33.57 ± 14.44	24.17 ± 13.93	0.837
Medical and family history^2^				
Family history of diabetes	21 (33.3%)	8 (38.1%)	13 (61.9%)	0.717
Newly diagnosed diabetes	39 (61.9%)	17 (43.6%)	22 (56.4%)	0.634
Recurrence of DKA (excluding current episode)	25 (39.7%)	9 (36%)	16 (64%)	0.491
Symptoms^2^				
Polyuria-polydipsia	41 (65.1%)	19 (46.3)	22 (53.7)	0.264
Polyphagia	22 (34.9%)	7 (31.8%)	15 (68.2%)	0.264
Weight loss	29 (46%)	12 (41.4%)	17 (58.6%)	0.987
Enuresis	12 (19%)	2 (16.7%)	10 (83.3%)	0.054
Abdominal pain	27 (42.9%)	11 (40.7%)	16 (59.3%)	0.941
Vomiting	29 (46%)	13 (44.8%)	16 (55.2%)	0.596
Drowsiness	19 (30.2%)	13 (68.4%)	6 (31.6%)	0.004*
Dyspnea	32 (50.8%)	19 (59.4%)	13 (40.6%)	0.003*
Physical examination^2^				
Tachycardia (age-dependent)	40 (63.5%)	19 (47.5%)	21 (52.5%)	0.120
Dehydration	46 (73%)	22 (47.8%)	24 (52.2%)	0.082
Cardiovascular collapse	8 (12.7%)	5 (62.5%)	3 (37.50%)	0.192
Triggering factors^2^				
Infection	22 (34.9%)	8 (36.4%)	14 (63.6%)	0.562
Insulin stopping	9 (14.3%)	6 (66.7%)	3 (33.3%)	0.095
Poor adherence to treatment	12 (19%)	6 (50%)	6 (50%)	0.495
Dietary non-compliance	15 (23.8%)	6 (40%)	9 (60%)	0.909
Laboratory results^1^				
WBC (×10^3 ^cells/mm³)	19 ± 7.5	25.13 ± 11.72	15.94 ± 8.60	0.013*
Na+ (mmol/L)	134.05 ± 5.67	134.15 ± 6.84	133.97 ± 5.00	0.137
K+ (mmol/L)	4.58 ± 1.10	3.94 ± 0.70	4.69 ± 1.20	0.291
BUN (g/L)	0.35 ± 0.15	0.38 ± 0.13	0.32 ± 0.16	0.805
serum creatinine (mg/L)	9.44 ± 4.73	11.07 ± 4.67	8.24 ± 4.65	0.528
PCT positive (ng/mL)	0.82 ± 2.16	1.68 ± 3.01	0.30 ± 1.21	0.011*
CRP (mg/L)	10.76 ± 20.80	16.63 ± 26.10	6.45 ± 14.98	0.009*
HbA1c (%)	11.61 ± 1.50	11 ± 1.34	12.57 ± 1.27	0.926
Treatment and outcome^1^				
Duration of insulin administration SAP (H)	37.57 ± 18.96	43.96 ± 19.32	33.08 ± 17.59	0.901
Duration of ICU stay (days)	3.44 ± 1.71	5.36 ± 2.26	4.67 ± 2.20	0.379
Duration of total hospitalization (days)	7.54 ± 3.45	7.03 ± 3.49	7.94 ± 3.41	0.678

Regarding medical history, no significant difference was found for the recurrence of DKA (p = 0.491) or a family history of diabetes (p = 0.717). Ketoacidosis was the first manifestation of diabetes in 27% of patients admitted for severe ketoacidosis and in 34.9% of those with non-severe ketoacidosis, but the difference was not significant (p = 0.634). The analysis regarding the symptoms showed a significant difference for drowsiness (p = 0.004) and dyspnea (p = 0.003), whereas the difference was not significant for the other symptoms, namely, polyuria-polydipsia syndrome (p = 0.264), polyphagia (p = 0.264), weight loss (p = 0.987), abdominal pain (p = 0.941), and vomiting (p = 0.351).

Regarding the biological parameters, a higher number of patients with severe DKA compared to those with non-severe DKA exhibited significantly higher levels of CRP (p = 0.009) and procalcitonin (p = 0.011), as well as significantly elevated WBC count (p = 0.013). In contrast, no significant differences were observed between the two groups regarding renal function parameters (urea and creatinine) or electrolyte levels (Na^+^ and K^+^). For triggering factors of DKA and the duration of hospitalization in both the intensive care unit and the hospital, no factors showed a statistically significant difference.

## Discussion

This study allowed us to identify certain factors influencing the severity of DKA in pediatric intensive care, both clinically and biologically. Clinically, the presence of dyspnea (p = 0.004) and altered consciousness (p = 0.003) at admission were significantly associated with severity. Biologically, a significant increase in WBC count (p = 0.013), CRP (p = 0.009), and PCT (p=0.038) were also linked to more severe DKA.

In our cohort, severe DKA accounted for 41.3% of cases, which is higher than the rates reported by Peng et al.(33.9%) [9] and Ongun et al. (35.2%) [10]. This variation could be due to differences in population characteristics or healthcare access. The average age in our study was similar to that observed in other studies [11,12], but no significant association was found between age, sex, weight, and DKA severity. These findings align with those of Blanc et al. [11], Hirschler et al. [13], Sehgal et al. [14], and Sayed et al. [15]. However, Razavi and Hamidi observed that DKA was more severe in females, which was not supported by our findings [16].

In 61.9% of our cases, DKA was the first manifestation of diabetes, indicating a delay in diagnosis. This finding is consistent with studies from Latin America (61%) [13] and Malaysia (67%) [16] but much higher than the 29.9% found in European countries [17]. We did not find any significant association between DKA severity and whether it was the first manifestation of diabetes [18], nor with a family history of diabetes [11], which aligns with other studies. Symptoms such as polyuria-polydipsia syndrome and weight loss were frequent but did not show a significant correlation with DKA severity. Vomiting and abdominal pain, although common, were also not linked to DKA severity. These results are consistent with the literature [13,16,19,20].

Regarding consciousness, a significant association was found between drowsiness at admission and DKA severity (p = 0.004), highlighting the importance of a thorough neurological assessment. This result is in line with the study by Syed et al. [15], which statistically demonstrated that the Glasgow Coma Scale score, as an indicator of consciousness, is related to DKA severity. It is critical to emphasize the need for close monitoring of neurological status in children, as they are more prone to developing severe neurological complications such as cerebral edema [7].

Dehydration was present in 73% of patients but did not show a significant correlation with DKA severity. Contradictory results are found in the literature on this topic, with Hirschler et al. [13] reporting a stronger link. Similarly, we did not find a statistically significant association between hemodynamic instability (tachycardia and cardiovascular collapse) and DKA severity. In the study by Ayed et al. [21], hypotension was considered a late and rare sign of severe DKA, underlining its potential importance. According to this study, patients with hypotension should be admitted to intensive care, which could suggest a correlation with more severe DKA.

Infections were a common triggering factor in our study, found in 34.9% of patients, but no statistically significant association was observed between infection and DKA severity (p = 0.562). Similarly, we did not find any significant link between poor treatment adherence and DKA severity, which contrasts with Syed et al. [21], who clearly indicated an association between infection, non-adherence to treatment, and DKA severity.

Concerning biological markers, the study found a significant correlation between elevated WBC counts, CRP, and PCT levels and the severity of DKA, while potassium and sodium levels did not show significant associations. The existing literature on these factors is contradictory, and we could not identify studies that compared severe DKA with biological parameters such as CRP and PCT. The study by Syed et al. [15] found no link between sodium and potassium levels and DKA severity. In contrast, the research by Razavi and Hamidi [16] and Abd El Salam Mohammed et al. [22] demonstrated a strong association between hypokalemia and the severity of DKA, while the study by Sehgal et al. [14] showed that WBC counts at admission were inversely correlated with serum pH, suggesting a relationship with DKA severity.

Limitations of the study

This study, while providing valuable insights into the factors associated with the severity of DKA in children admitted to pediatric intensive care, has several limitations that should be noted. First, the retrospective nature of the study introduces inherent limitations regarding the quality and availability of data, which may have affected the analysis of certain factors, such as detailed medical history and treatment adherence. Second, the relatively small sample size (63 patients) limits the statistical power of the analysis. Third, as the study was conducted in a single center, the results may not be generalizable to other hospitals or regions, especially in rural areas where access to care may be more limited. Finally, the lack of long-term follow-up of the patients prevents the assessment of the long-term impact of DKA episodes on the progression of diabetes in these children, as well as the estimation of the risk of recurrence.

## Conclusions

This study allowed us to identify certain factors associated with the severity of DKA. Statistically significant correlations were observed between DKA severity and factors such as the clinical presence of dyspnea and neurological disturbances at admission, as well as biological markers of an inflammatory syndrome, including elevated WBC count, CRP, and PCT levels. This study provides valuable insights into the factors influencing the severity of DKA in children in Morocco. Understanding these factors could help clinicians provide better management, improving the prognosis of this condition. Given the small size of our series, a multicenter study with a larger sample would provide more robust data and statistical power, potentially identifying additional factors related to the severity of this complication.
